# Biological properties of Ceraputty as a retrograde filling material: an in vitro study on hPDLSCs

**DOI:** 10.1007/s00784-023-05040-z

**Published:** 2023-05-01

**Authors:** Sergio López-García, Francisco J. Rodríguez-Lozano, José Luis Sanz, Leopoldo Forner, María Pilar Pecci-Lloret, Adrián Lozano, Laura Murcia, Sonia Sánchez-Bautista, Ricardo E. Oñate-Sánchez

**Affiliations:** 1 Departament d’Estomatologia, Facultat de Medicina I Odontologia, Universitat de València, 46010 Valencia, Spain; 2 Department of Dermatology, Stomatology, Radiology and Physical Medicine, Morales Meseguer Hospital, Faculty of Medicine, University of Murcia, 30008 Murcia, Spain; 3 Department of Stomatology, Faculty of Medicine and Dentistry, Universitat de València, C/ Gascó Oliag 1, 46010 Valencia, Spain; 4 Department of Health Sciences, Catholic University San Antonio of Murcia, 30107 Murcia, Spain

**Keywords:** Bioactivity, Biocompatibility, Biodentine, Ceraputty, Endodontics, Endosequence root repair material, Periodontal ligament stem cells

## Abstract

**Objectives:**

To assess the cytocompatibility and bioactive potential of the new calcium silicate-based cement Ceraputty on human periodontal ligament stem cells (hPDLSCs) compared to Biodentine and Endosequence BC root repair material (ERRM).

**Materials and methods:**

hPDLSCs were isolated from extracted third molars from healthy donors. Standardized sample discs and 1:1, 1:2, and 1:4 eluates of the tested materials were prepared. The following assays were performed: surface element distribution via SEM–EDX, cell attachment and morphology via SEM, cell viability via a MTT assay, osteo/cemento/odontogenic marker expression via RT-qPCR, and cell calcified nodule formation via Alizarin Red S staining. hPDLSCs cultured in unconditioned or osteogenic media were used as negative and positive control groups, respectively. Statistical analysis was performed using one-way ANOVA or two-way ANOVA and Tukey’s post hoc test. Statistical significance was established at *p* < 0.05.

**Results:**

The highest Ca^2+^ peak was detected from Biodentine samples, followed by ERRM and Ceraputty. hPDLSC viability was significantly reduced in Ceraputty samples (*p* < 0.001), while 1:2 and 1:4 Biodentine and ERRM samples similar results to that of the negative control (*p* > 0.05). Biodentine and ERRM exhibited an upregulation of at least one cemento/odonto/osteogenic marker compared to the negative and positive control groups. Cells cultured with Biodentine produced a significantly higher calcified nodule formation than ERRM and Ceraputty (*p* < 0.001), which were also higher than the control groups (*p* < 0.001).

**Conclusion:**

Ceraputty evidenced a reduced cytocompatibility towards hPDLSCs on its lowest dilutions compared to the other tested cements and the control group. Biodentine and ERRM promoted a significantly higher mineralization and osteo/cementogenic marker expression on hPDLSCs compared with Ceraputty. Further studies are necessary to verify the biological properties of this new material and its adequacy as a retrograde filling material.

**Clinical relevance:**

This is the first study to elucidate the adequate biological properties of Ceraputty for its use as a retrograde filling material.

## Introduction

The widely ranged applications of calcium silicate–based cements (CSCs) in clinical endodontics have resulted in the development of new materials with improved physical–mechanical and biological properties. The enhancement of such properties can be achieved via the modification of their essential formulation or the inclusion of additives in their composition [1, 2]. Within the field of microsurgical endodontics, root repair or “putty” CSCs can be applied as retrograde filling materials [3, 4].

Traditionally, mineral trioxide aggregate (MTA), glass ionomers, super ethoxy benzoic acid (super EBA), or dental amalgam were proposed as retrograde filling materials. Particularly, MTA-based formulations gained popularity in this regard. However, they present several disadvantages, such as difficult handling and discoloration potential [5, 6]. With the introduction of CSCs, categorized as bioactive materials, traditional materials are being displaced [7]. Several CSCs have been investigated in the last years and have demonstrated their numerous advantages. Nevertheless, so far, no specific material has been developed that meets all the desirable properties for their application in microsurgical endodontics [8, 9].

In 2009, Biodentine™ (Septodont, Saint Maur des Fossés, France) was introduced in the market to solve the drawbacks of MTA: tooth discoloration potential, extensive setting time, complex handling, and high cost [10, 11]. Although reports suggest using Biodentine as an alternative to MTA-based cements, there is not sufficient evidence to confirm the clinical superiority of Biodentine as a root-end filling material in endodontic microsurgery [12]. A newer premixed CSC, namely EndoSequence root repair material (ERRM) (Brasseler USA, Savannah, GA, USA), has been recently investigated as a root-end filling material, root repair material, pulp capper, and apical plug for apexification procedures. Previous reports considered ERRM as a suitable material to achieve an effective seal of the root canal system thanks to its biomineralization potential [13-15]. Similarly, Ceraputty (Meta Biomed Co., Cheongju, Korea) is a newly launched premixed endodontic cement containing calcium silicates, zirconium oxide, and a thickening agent. To date, however, there is no published evidence on the biological properties of this new CSC.

Periapical tissue’s reaction is related with healing prognosis [16]. Periodontal ligament stem cells (PDLSCs) are a subgroup of dental stem cells (DSCs) with a multilineage differentiation potential and osteo/odonto/cementogenic differentiation potential [17]. They are located within the perivascular space of the periodontium and thus will be in direct contact with the root-end filling material [18]. For this reason, numerous in vitro studies have used PDLSCs as target cell lines to preliminarily assess the biological properties of new endodontic materials, such as endodontic sealers and root repair materials [19].

Within this framework, this study aimed to compare the biological properties of the newly introduced CSC Ceraputty compared to two established CSCs, ERRM, and Biodentine, by the assessment of the cytotoxicity and biomineralization ability of the three materials. The null hypothesis was that the three tested CSCs would show no differences in their cytotoxicity or biomineralization ability.

## Material and methods

The manuscript of this laboratory study has been written following the “Guidelines for reporting pre-clinical in vitro studies on dental materials” [20].

### Cell isolation, culture, and characterization

The protocol to obtain human PDLSCs (hPDLSCs) was approved by the Ethics Committee of University of Murcia (Murcia, Spain; IRB number 2199/2018). Written informed consent was obtained from healthy donors (*n* = 10, 18–23-years old), who provided molars (*n* = 10) from which to isolate hPDLSCs and agreed to their use in this study. The sample size was calculated using openepi software (www.openepi.com), with a confidence interval of 95% and a power of 80%, and was based on the methodology of a previous similar study [21]. Patient data and images that could be used to identify the study participants were not included in this work. Periodontal tissues were aseptically removed and immersed for incubation in 0.25% trypsin in 4 mL of EDTA (Life Technologies, USA) for 30 min at 37 °C. After neutralizing them with 4 mL of medium, the cells were detached by pipetting the solution, and then they were filtered via a strainer (70 μm, Corning, USA). The hPDLSCs were cultured in Dulbecco’s modified Eagle’s medium containing 10% fetal bovine serum, penicillin (100 U/mL), and streptomycin (100 μg/mL) (Gibco, Life Technologies) supplemented with 100 mM ascorbic acid (Sigma) in a 37 °C incubator with 5% CO2 (Thermo Forma 3110, Thermo Fisher, USA).

Previously characterized hPDLSCs were used for the subsequent biological assays. The characterization process followed the International Society of Cellular Therapy (ISCT) guidelines, as follows: The overexpression of mesenchymal stem cell (MSC)-specific surface markers and the low rexpression of hematopoyetic markers were confirmed via flow cytometry (FACSCalibur Flow Cytometry System; BD Biosciences). The selection of markers was performed based on previous similar studies [22, 23]. Lastly, the trilineage mesenchymal differentiation potential was confirmed via their culture in osteogenic, chondrogenic, and adipogenic media (Miltenyi Biotec). hPDLSCs of passages 3 to 5 were used in this study.

### Material extract preparation

Biodentine, Ceraputty, and Endosequence root repair material were tested in this study (Table 1). Cylindrical rubber molds (*n* = 30) were prepared with a diameter of 5 mm and height of 2 mm and were disinfected by exposure to UV light for 30 min. The number of discs was based on the protocol from a previous study with similar methodology [24]. CSMs were mixed according to their respective manufacturer’s instructions and allowed to set for 24 h. Each disc was placed in a separate well from a 24-well plate and immersed in a fresh growth medium for 24 h at 37 °C. The extraction was performed in accordance with ISO 10993–5 [25]. Thereafter, to study the effect of the concentration of each material on their biological properties, various dilutions (1:1, 1:2 and 1:4 v/v) of these extraction media were prepared using fresh complete DMEM [24]. The following in vitro biological assays were performed by a single operator.

**Table 1:** Tested materials

Materials	Manufacturer	Composition	Lot number
CeraPutty Bioceramic root canal filling material	Meta Biomed Co., 270, Osongsaengmyeong 1-ro, Osong-eup, Heungdeok-gu, Cheongju-si, Chungcheongbuk-do, South Korea	Zirconium dioxide, tricalcium silicate, dicalcium silicate, tricalcium aluminate	**CPT2111231**
Endosequence BC RRM Putty	BUSA. Innovative Bioceramix Inc. 101–8218 North Fraser Way Burnaby, BC V3N 0E9, Canada	Zirconium oxide, tricalcium silicate, dicalcium silicate, calcium sulfate, tantalum peroxide	**2102BPP**
Biodentine	SEPTODONT. 58, rue du Pont de Créteil 94,107 Saint-Maur-des-Fossés Cedex, France	Tricalcium silicate, zirconium oxide, calcium oxide, calcium carbonate and colorings. Aqueous solution consisting of calcium chloride and polycarboxylate	**B28914**

### Cell viability assay

The cytotoxicity of the three CSM eluates was evaluated using an MTT (3-(4,5-dimethylthiazol-2-yl)-2,5-diphenyltetrazolium bromide) assay, as previously reported by similar studies [26]. For this purpose, 4 × 10^3^ cells were seeded into 96-well culture plates. After 1, 2, and 3 days of culture with the material eluates, an MTT reagent (Sigma Aldrich) was added for 4 h, following its manufacturer’s instructions. When a purple precipitate was detectable, dimethylsulfoxide (DMSO) (Sigma-Aldrich) was added to each well (100 μl/well), and plates were covered and kept in dark conditions for 4 h to solubilize the formazan crystals produced by viable cells, after reducing the MTT reagent. Finally, absorbance was measured at 570-nm wavelength using a microplate reader (Synergy H1, BioTek,Winooski, VT, USA).

### Scanning electronic microscopy

hPDLSCs (5 × 10^3^ cells/well) were cultured on disinfected tested material disks in 48-well plates and incubated at 37 °C for attachment in a 5% CO_2_ incubator during 72 h. The cements with the hPDLSCs were washed with PBS thrice and were exposed to 4% glutaraldehyde (Sigma-Aldrich) solution for fixation. Then, the samples were washed with PBS thrice and serially dehydrated with ethanol and treated with hexamethyldisilazane (Sigma-Aldrich) for 5 min. The dehydrated samples were coated with gold and palladium and examined via SEM (Jeol 6100 EDAX; Jeol Inc.) at 100 × , 300 × , and 1500 × magnifications. In addition, the superficial chemical composition, and morphological properties of CSMs were assessed by energy-dispersive X-ray spectroscopy (EDX).

### RT-qPCR gene expression analysis

To determine mRNA transcript levels of differentiation and mineralization markers, hPDLSCs were cultured 21 days in undiluted (1:1) conditioned media from the three CSMs, in unconditioned culture media (negative control groups), or in osteogenic differentiation media (positive control; OsteoDiff media; Miltenyi Biotec). Culture media with fresh eluates from the respective groups were replaced every 3 days as previously reported by our research group [24].

The primer sequences for the differentiation markers used for the assay were as follows (5′–3′): Cementum attachment protein or CAP (forward: TTTTTCTGGTCGCGTGGACT, reverse: TCACCAGCAACTCCAACAGG), cementum protein 1 or CEMP1 (forward: GGGCACATCAAGCACTGACAG, reverse: CCCTTAGGAAGTGGCTGTCCAG), alkaline phosphatase or ALP (forward: TCAGAAGCTCAACACCAACG, reverse: TTGTACGTCTTGGAGAGGGC), runt-related transcription factor 2 or RUNX2 (forward: TCCAC ACCATTAGGGACCATC, reverse: TGCTAATGCTTCGT GTTTCCA), bone sialoprotein or BSP (forward: TGCC TTGAGCCTGCTTCCT, reverse: CTGAGCAAAATTAA AGCAGTCTTCA), amelogenin X, or AMELX (forward: CACCCTGCAGCCTCATCACC, reverse: GTGTT GGATTGGAGTCATGG).

Differentiation marker expression was measured relative to the expression of the housekeeping gene glyceraldehyde 3-phosphate dehydrogenase (GAPDH), with the following sequence (5′-3′): (forward: TCAGCAATGCCTCCTGCAC, reverse: TCTGGG TGGCAGTGATGG). To calculate the relative gene expression, the standardized 2 − ΔΔCT method was used [27].

### Alizarin Red S staining

Mineralization or calcification ability of the tested CSMs on hPDLSCs were evaluated by Alizarin Red S staining after 21 days of culturing with undiluted (1:1) cement-conditioned medium, as follows: (A) control (DMEM), (B) Osteodiff, (C) Ceraputty, (D) ERRM, and (E) Biodentine. After the culture period, the samples were rinsed with fetal bovine serum and fixed with 70% ethanol for 1 h. Then, samples were stained with 2% Alizarin Red solution (Sigma Aldrich) for 30 min in controlled conditions (dark ambient and room temperature) and solubilized using 10% cetylpyridinium chloride monohydrate solution (Sigma-Aldrich). Finally, Synergy H1 multi-mode microplate reader (BioTek, Winooski, VT, USA) was used to measure the absorbance values of the samples at 570 nm. The methodology for Alizarin Red S staining assay was based on a previous similar study [21].

### Statistical analysis

All assays which were statistically assessed were performed three times. For quantification, data were calculated as means and standard deviations (SDs). The normality in the distribution of the data was previously confirmed via a Q-Q plot. Statistical significance was tested using one way ANOVA (MTT assay) or two-way ANOVA (ARS assay; RT-qPCR assay) and Tukey’s post hoc test using Graph-Pad Prism v8.1.0 (GraphPad Software). Each dilution was considered an independent treatment. Here, * indicates a *P* value below 0.05, ** indicates a *P* value below 0.01, and *** indicates a *P* value below 0.001.

## Results

### MTT assay

**Fig. 1:**
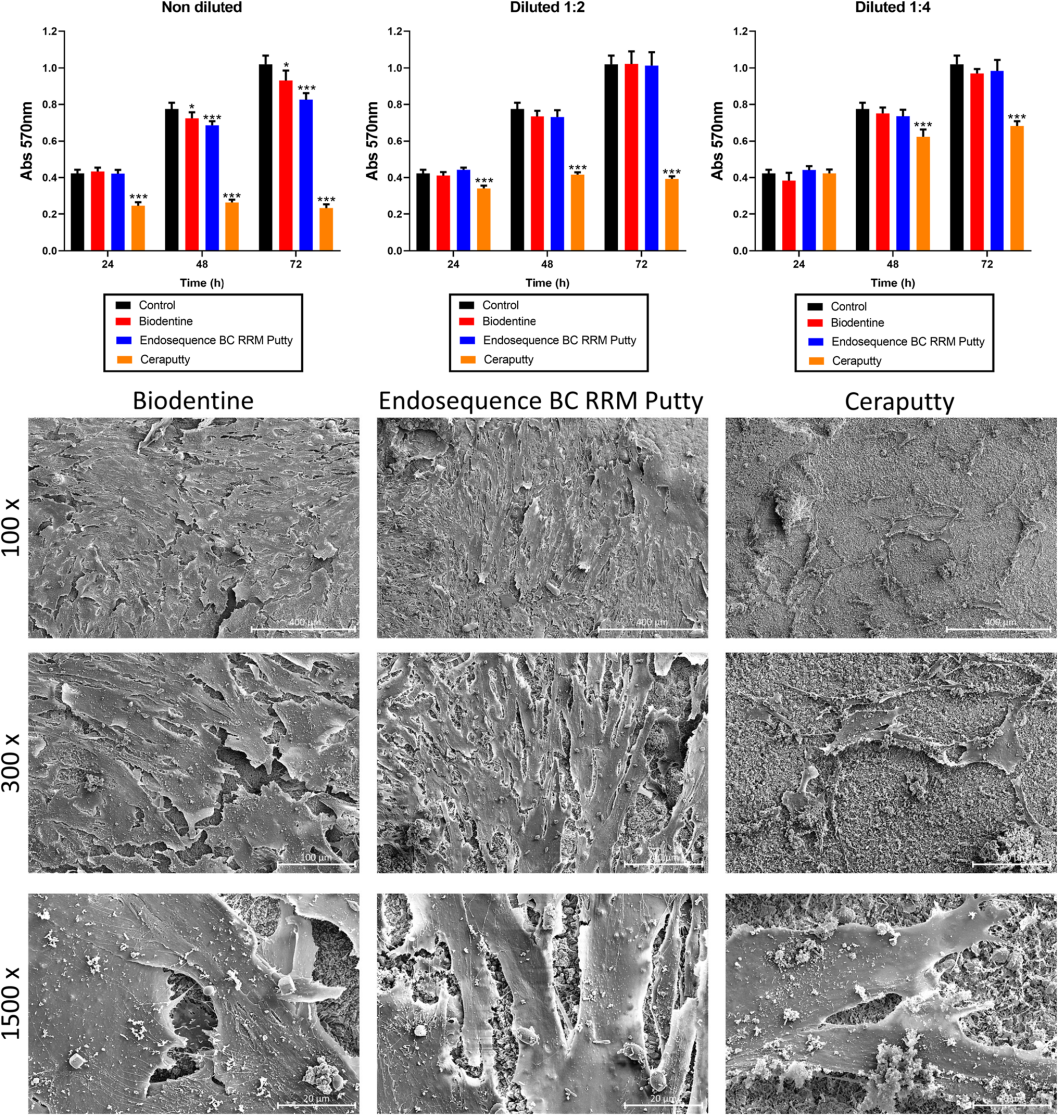
**A** MTT assay results after 24, 48, and 72 h of culture with hPDLSCs. Results are divided by material (Ceraputty, Biodentine, ERRM) and eluate (1:1; 1:2; 1:4) and compared to a negative control group (hPDLSCs cultured in unconditioned medium). Absorbance: 570 nm. One-way ANOVA analysis (*p < 0.05. ***p < 0.001). **B** Representative SEM images of the results of the adhesion and morphology assay after 72 h of culture of hPDLSCs seeded onto the surface of the tested materials (Ceraputty, Biodentine, ERRM). Scale bars: 400 μm at 100 × magnification; 100 μm at 300 × magnification; 20 μm at 500 × magnification

The MTT assay revealed an adequate cell viability from all hPDLSCs cultured with 1:2 and 1:4 eluates of Biodentine and ERRM at all the tested time points (24, 48, and 72 h of culture), similar to that of the control group. Undiluted Biodentine and ERRM-treated cells, however, showed slight decreases in viability compared with the control group (*p* < 0.05 and *p* < 0.001, respectively). Ceraputty-treated cells exhibited a moderately lower viability compared with the untreated group (control) after all time-points (*p* < 0.001; Fig. 1A).

### Scanning electronic microscopy

hPDLSCs seeded on Biodentine and ERRM showed a better cell adhesion process in terms of substrate attachment, spreading, and cytoskeleton development on the niche-like structures of the cement than Ceraputty, which evidenced a moderate quantity of cells and less cytoplasmatic interaction between cells (Fig. 1B).

**Fig. 2:**
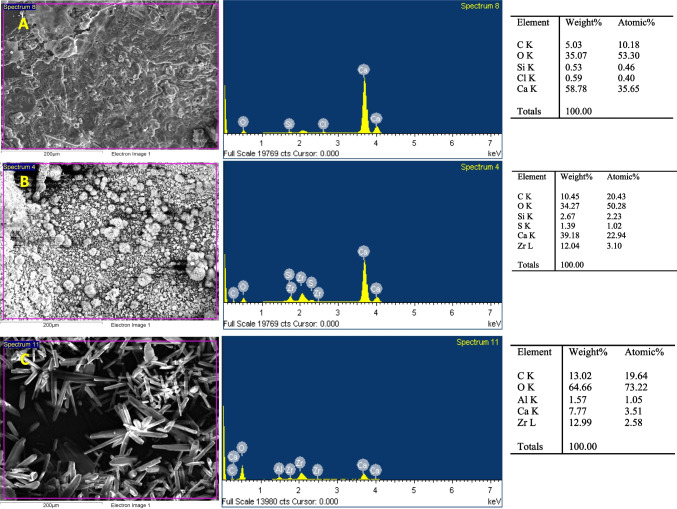
SEM–EDX results. First row: SEM images of each material (scale bar: 200 μm). Second row: EDX elemental spectra. Third row: elements present per cement by weight and atomic weight. **A** Biodentine, **B** ERRM, **C** Ceraputty

The EDX analysis provided the qualitative and semi-quantitative superficial elemental composition of each cement, which are represented in Fig. 2. SEM micrographs evidenced that the particle sizes and shapes differed between the tested materials. Ceraputty exhibited a large number of petal-like crystallized structures whereas the particle size and shape were more homogeneous in Biodentine. Peaks of Zr were detected in both ERRM and Ceraputty with similar percentages. With regards to Ca^2+^, Biodentine contained a higher percentage of Ca^2+^ compared with ERRM and Ceraputty. On the other hand, the percentage of Si in ERRM was higher compared with Biodentine.

### RT-qPCR analysis

**Fig. 3:**
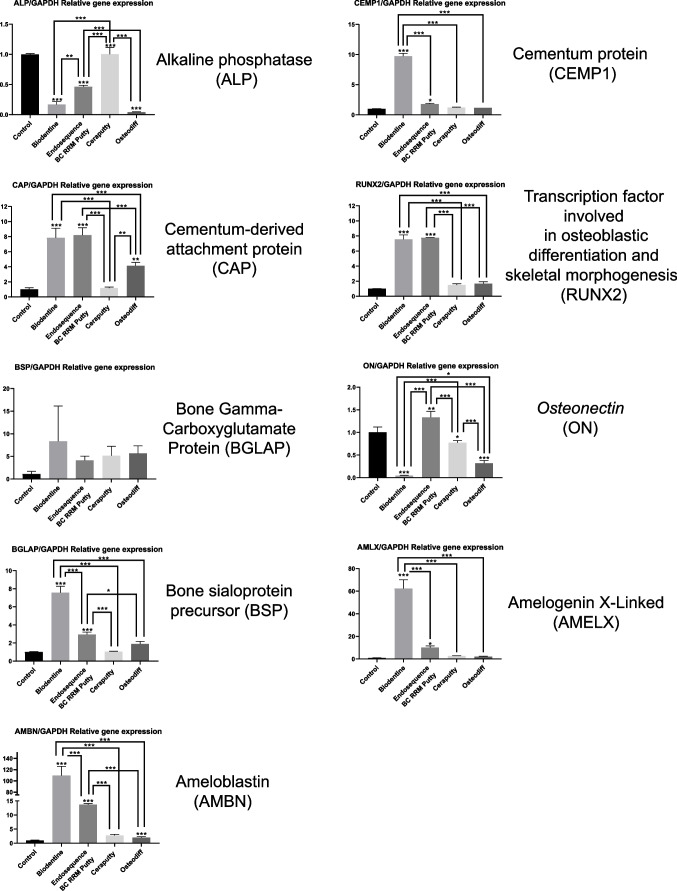
RT-qPCR results. hPDLSC marker expression after 21 days of culture in unconditioned culture media (negative control), osteogenic culture media (positive control), or with the tested materials (Ceraputty, Biodentine, ERRM). Two-way ANOVA analysis (*p < 0.05. **p < 0.01. ***p < 0.001)

The evaluation of the differentiation ability of the hPDLSCs cultured with the cement eluates were performed using RT-qPCR (Fig. 3). ALP expression decreased in hPDLSCs exposed to the tested materials and Osteodiff group (negative control) for 21 days, probably related to the early expression of osteoblastic differentiation. On the other hand, the expression of the genes CEMP, BSP, AMELX, and AMBN induced by Biodentine was significantly higher than the other groups (***p < 0.001). hPDLSCs exposed to Biodentine and ERRM produced a few folds more of CAP and RUNX2 genes than the other groups. Finally, the expression of ON, was also highly expressed in hPDLSCs exposed to ERRM (**p < 0.01) compared to the untreated group. Ceraputty, on the other hand, did not show a significant upregulation of any of the tested markers.

### Alizarin Red S staining assay

**Fig. 4:**
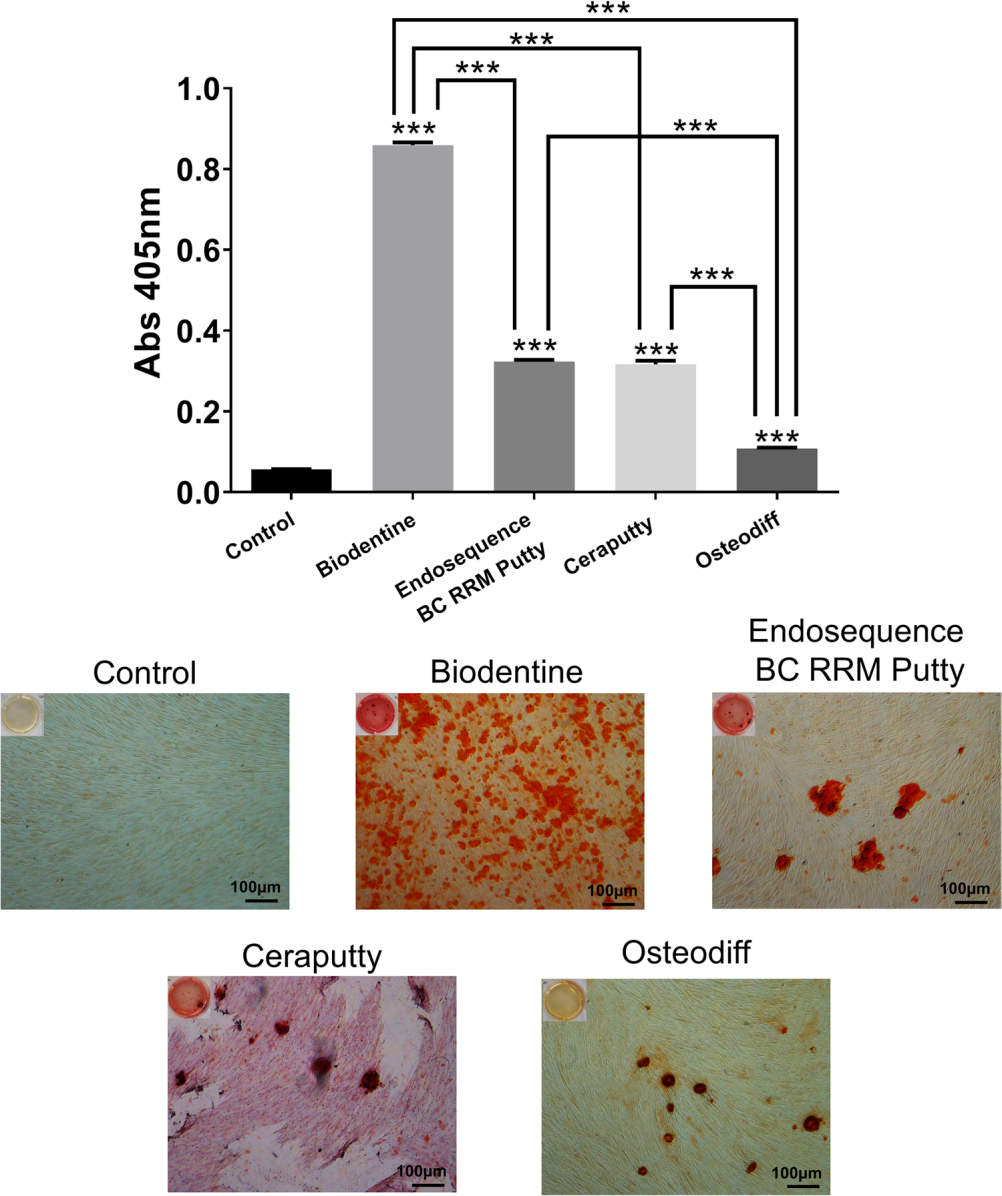
ARS mineralization assay results after 21 days of culture of hPDLSCs in unconditioned culture media (negative control), osteogenic culture media (positive control), or with the tested materials (Ceraputty, Biodentine, ERRM). Two-way ANOVA analysis (***p < 0.001)

Alizarin Red S Staining (ARS) serves as a marker of mineralization and osteo/cementogenesis by detecting the formation of calcified nodules. As shown in Fig. 4, more pronounced calcium deposits were observed in the tested groups compared with the control and Osteodiff groups (***p < 0.001). Meanwhile, the results showed deeper staining with undiluted concentrations of Biodentine (***p < 0.001). These data suggest that Biodentine could significantly promote the osteo/cementogenic differentiation of hPDLSCs.

## Discussion

An ideal endodontic retrograde filling material should present adequate biological properties that favor the healing process of existing periapical lesions and/or prevent the appearance of new lesions. The bioactive potential, i.e., the ability of a material to form a superficial mineralized attachment to the inorganic component of the dentin substrate, is closely related to the material’s biocompatibility and chemical composition [28, 29]. The modification in the composition of new endodontic materials could lead to differences in their clinical behavior. For example, it has been studied that even the differences in the radiopacifying agent can affect the biological properties of CSCs, among others [29]. Within this framework, the aim of this study was to assess the cytocompatibility and bioactive potential of the Ceraputty and to compare them to that exhibited by the Biodentine and ERRM.

As mentioned previously, root repair materials should provide a favorable environment for periapical tissue repair. For this reason, they cannot alter the proper functioning and the viability of periapical cells negatively [30]. Altogether, available evidence highlights the importance of hPDLSCs in the maintenance of homeostasis of the periodontium and its repair [31, 32]. Thus, they were selected as the target cells for this laboratory study. The alternative use of immortalized or animal cells was discarded, since they are more resistant that human cells and may show different sensitivities to the tested materials [33].

The cell viability rates of Ceraputty samples, although significantly reduced (*p* < 0.001), were higher in the 1:4 dilution, evidencing a moderate metabolic activity that suggests a moderate cell proliferation. Previous studies have also reported the increased cytocompatibility of CSCs as more diluted [34]. In the present study, the use of several dilutions (1:1, 1:2, and 1:4) was performed to better predict the clinical conditions, in which the tested materials can be placed on dentin thicknesses of 0.01 to 0.25 mm or directly on periapical tissues.

Cell attachment onto the surface of biomaterials is another indicator of their biocompatibility, since cells in contact with a biomaterial may be directly affected if the material releases cytotoxic components [35, 36]. hPDLSCs were shown to fully spread and attach over the surface of set Biodentine and ERRM samples (Fig. 1B–E). Previous studies have also shown the favorable attachment properties of hPDLSCs to CSCs [15, 22]. However, in accordance with MTT assay, moderate cell attachment was observed on Ceraputty’s surface. As a limitation of the present study, it should be highlighted that the absence of information on the presence or absence of thickening agents, additives, fillers, and/or vehicles can act as a limitation of the analysis of the cytocompatibility of the tested materials from the perspective of their composition.

Many studies have described the regulatory effect of calcium silicate on hPDLSCs, which may be related to the leaching of ions from these materials [36, 37]. EDX analysis revealed a higher peak of Ca^2+^ in Biodentine compared with ERRM and Ceraputty. Nevertheless, a limitation of EDS analysis is that it cannot identify calcium hydroxide peaks and other crystalline phases in hydraulic cements after setting. For this reason, other complementary techniques could be of use, such as attenuated total reflection–Fourier transform infrared (ATR-FTIR) spectroscopy or X-ray diffraction (XRD) analysis [38]. The high percentage of calcium ions in Biodentine may act as an explanation for the significantly higher mineralization evidenced by Biodentine-treated cells compared with those treated with ERRM or Ceraputty in the ARS assay.

Regarding RT-qPCR assay, a general pattern was evidenced. Following the methodology of previous studies, CEMP1, CAP, ALP, RUNX2, ON, BGLAP, BSP, AMBN, and AMELX were used as markers for osteo/cementogenic differentiation [37, 39, 40]. As expected, Biodentine-treated cells showed a significant upregulation of the majority of markers compared with the untreated cells and the positive control group, in agreement with the previous biological assays. Following the same pattern, a previous report showed the overexpression of osteogenic markers in the presence of other hydraulic cements such as Biodentine, Bioaggregate (Innovative Bioceramix, Vancouver, BC, Canada), and ProRootMTA (Dentsply, Tulsa, USA) [41]. Our research group also demonstrated that TotalFill BC RRM putty (FGK, Dentaire SA, La-Chaux-de-fonds, Switzerland), induced RUNX2, CAP, and CEMP1 overexpression in hPDLSCs [24].

Lastly, the mineralization ability is a crucial parameter to evaluate the bioactive properties of endodontic biomaterials [35]. An increased production of calcified nodules was observed in Biodentine-treated cells compared with ERRM and Ceraputty groups in the ARS assay, which indicated that Biodentine may effectively promote osteo/cementogenic differentiation of hPDLSCs. Similar results have been observed in previous studies, in which putty calcium silicate materials induced calcified nodule formation [24, 42].

Previous studies have reported differences between the biological properties of set and freshly mixed endodontic biomaterials [43]. In the present study, the materials were tested after setting, as performed in previous studies [34, 44]. Nevertheless, future studies could assess the cytocompatibility and/or bioactivity of endodontic biomaterials in different conditions, such as setting or pH variations [45]. Even variations in the target cell lines could be assessed, such as the use of DSCs from donors with underlying pathologies or inflammatory DSCs [46]. This could be relevant since, as highlighted by a recent study, differences can be found not only between DSC subtypes, but within a same subtype [47]. For this reason, it is also crucial to characterize the cell populations to be used in this type of studies [48].

To the authors’ knowledge, this is the first study to elucidate the biological properties of the new CSC Ceraputty. Both cytocompatibility and bioactivity assays were used to provide a wide spectrum of data regarding the tested materials. These two factors, together with the use of standardized methodology for sample preparation (ISO 10993–5 guidelines) and data reporting (Guidelines for reporting pre-clinical in vitro studies on dental materials) [20] can be considered as the strengths of the present study. However, the inherent in vitro nature of this study can be highlighted as its main limitation.

For this reason, the data reported should be interpreted with caution and treated as a preliminary assessment, which should be complemented and contrasted with future studies on animal and/or human models. Additionally, further in vitro studies should assess the physical–mechanical properties of Ceraputty, i.e., handling, setting, elemental characterization…in order to provide a better picture of the suitability of this cement as a retrograde filling material.

## Conclusions

In the present study, the new calcium silicate cement-based material Ceraputty evidenced a reduced cytocompatibility towards hPDLSCs on its lowest dilutions compared to the other tested cements and the control group. Parallely, Biodentine and ERRM promoted a significantly higher mineralization and osteo/cementogenic marker expression on hPDLSCs compared with Ceraputty. Further studies are necessary to verify the biological properties of this new material and its adequacy as a retrograde filling material.

## Data Availability

The data presented in this study are available on request from the corresponding author.
